# Lack of aminoacids in mouse hepatocytes in culture induces the selection of preneoplastic cells

**DOI:** 10.1186/1753-6561-6-S3-P13

**Published:** 2012-06-01

**Authors:** Andrea N Chisari, Stella M Echarte, Enrigue Podaza, Isabel Fabregat

**Affiliations:** 1 Biological Research Institute y Chemical Department, Mar del Plata National University, Mar del Plata, CP7600, Argentina; 2 Biological Clues of the Invasive and Metastatic Phenotype Group, Bellvitge Biomedical Research Institut (IDIBELL), L’Hospitalet de Llobregat, Barcelona, Spain

## 

Protein malnutrition occurs when there is insufficient protein to meet metabolic demands. Previous works have indicated that cycles of protein fasting/refeeding enhance the incidence of early lesions during chemical carcinogenesis in rat liver. In mammals, protein malnutrition causes high susceptibility to infection diseases and cancer. In liver, both caloric and proteic malnutrition cause severs metabolic changes [1].

*The general objective* of this work was to study the effect of aminoacids (Aa) deprivation on the proliferation and survival of hepatocytes, to understand its possible involvement in the generation of preneoplastic stages in the liver.

*Experimental procedures:* cell line derived from newborn mice hepatocytes (Parental cells=Par) [2] were cultured in complete medium, or aminoacid-starved medium (PM=private medium).

When hepatocytes are cultured in the absence of Aa, the cells detaches and die through apoptosis. After 72 h of culture with PM the few surviving cells were incubated with complete medium. After changing the medium, cells began to proliferate and expanded (Selected Cells=Sel).

Sel cells showed a significant higher proliferation rate than the Par ones. This conclusion was confirmed by studying the incorporation of [3H]-Thymidine as an analysis of DNA synthesis. In the presence of different concentrations of Fetal Bovine Serum, indicating that response to extracellular mitogens is enhanced in these cells. The response to the Transforming Growth Factor-beta (TGF-β), a physiological inducer of hepatocyte apoptosis whose concentration is elevated in liver tumors to counteract the abnormal growth of preneoplastic cells [3,4][5], is altered in Sel cells. Both TGF-β-induced decrease in cell viability and caspase-3 activation were attenuated in Sel when compared to the parental cells.

Both p-ERKs and p-AKT levels were much higher in Sel cells than in the Par ones. Downstream AKT signals, such as p70S6 and GSK3 were also higher phosphorylated in Sel cells. Interestingly, showed increased levels of p-SRC, which correlated with increase in the levels of phosphorylation of the EGF receptor (EGFR). These results indicate that the EGFR/SRC pathwaymight be overactivated in Sel cells. Sel cells expressed higher levels of EGFR ligands, such as TGF-α and HB-EGF; semiquantitative RT-PCR analysis clearly revealed that both ligands were overexpressed in Sel cells, when compared with normal hepatocytes. Moreover it is noteworthy that MAPK proteins c-JNK and p38, which remained increased in Sel cells.

*In conclusion*, results presented indicate that it is possible to isolate in vitro a population of hepatocytes that are able to survive in the absence of Aa, which has higher capacity to proliferate, showing a preneoplastic phenotype. This could explain why the alternately deprivation of proteins in diet could induce hepatocarcinogenesis susceptibility.
